# Telemedicine interventions in six conflict-affected countries in the WHO Eastern Mediterranean region: a systematic review

**DOI:** 10.1186/s13031-022-00493-7

**Published:** 2022-12-14

**Authors:** Pylin Parkes, Timesh D. Pillay, Yamama Bdaiwi, Remi Simpson, Nadim Almoshmosh, Lina Murad, Aula Abbara

**Affiliations:** 1 St Marys Hospital, Imperial College, London, W2 1NY UK; 2 King’s College, London, UK; 3 NAFS Health & Wellness Clinic, Bury St Edmunds, UK; 4 Metropolitan Access Centre, Washington, DC USA; 5 Syria Public Health Network, London, UK

**Keywords:** Telemedicine, Telehealth, Conflict, Syria, Afghanistan, Iraq, Libya, Yemen, Gaza, Tele-mental health

## Abstract

**Background:**

The COVID-19 pandemic has escalated the use of telemedicine in both high and low resource settings however its use has preceded this, particularly in conflict-affected settings. Several countries in the WHO Eastern Mediterranean (EMR) region are affected by complex, protracted crises. Though telemedicine has been used in such settings, there has been no comprehensive assessment of what interventions are used, their efficacy, barriers, or current research gaps.

**Main body:**

A systematic search of ten academic databases and 3 grey literature sources from January 1st 2000 to December 31st 2020 was completed, identifying telemedicine interventions in select EMR conflict-affected settings and relevant enablers and barriers to their implementation. Included articles reported on telemedicine use in six conflict-affected EMR countries (or territories) graded as WHO Health Emergencies: Afghanistan, Gaza, Iraq, Libya, Syria and Yemen. Data were extracted and narratively synthesised due to heterogeneity in study design and outcomes. Of 3419 articles identified, twenty-one peer-reviewed and three grey literature sources met the inclusion criteria. We analysed these by context, intervention, and evaluation. Context: eight related to Afghanistan, eight to Syria and seven to Iraq with one each in Yemen and Gaza. Most were implemented by humanitarian or academic organisations with projects mostly initiated in the United States or Europe and mostly by physicians. The in-country links were mostly health professionals rather than patients seeking specialist inputs for specialities not locally available. Interventions: these included both SAF (store and forward) and RT (real-time) with a range of specialities represented including radiology, histopathology, dermatology, mental health, and intensive care. Evaluation: most papers were observational or descriptive with few describing quality measures of interventions.

**Conclusions:**

Telemedicine interventions are feasible in conflict-affected settings in EMR using low-cost, accessible technologies. However, few implemented interventions reported on evaluation strategies or had these built in. The ad hoc nature of some of the interventions, which relied on volunteers without sustained financial or academic investment, could pose challenges to quality and sustainability. There was little exploration of confidentiality, ethical standards, data storage or local healthcare worker and patient acceptability.

**Supplementary Information:**

The online version contains supplementary material available at 10.1186/s13031-022-00493-7.

## Introduction

Protracted, complex armed conflicts have adverse effects on population health and on local health systems; these include damage to healthcare infrastructure and the deaths or exodus of healthcare workers leaving gaps in the workforce [1, 2]. This has increasingly detrimental effects on population health, particularly where the most experienced or specialised healthcare workers have been forcibly displaced with consequent effects on the education and training of more junior healthcare workers [1, 3]. Telemedicine can provide an opportunity for remote support where there is a dearth of healthcare workers or where specialist inputs are required. It can provide an innovative, low-cost, consistent mode of support in settings affected by conflict; however, implementation in such settings can be affected by a lack of infrastructure, connectivity, local human resources and financial investment [4].

**Fig. 1:**
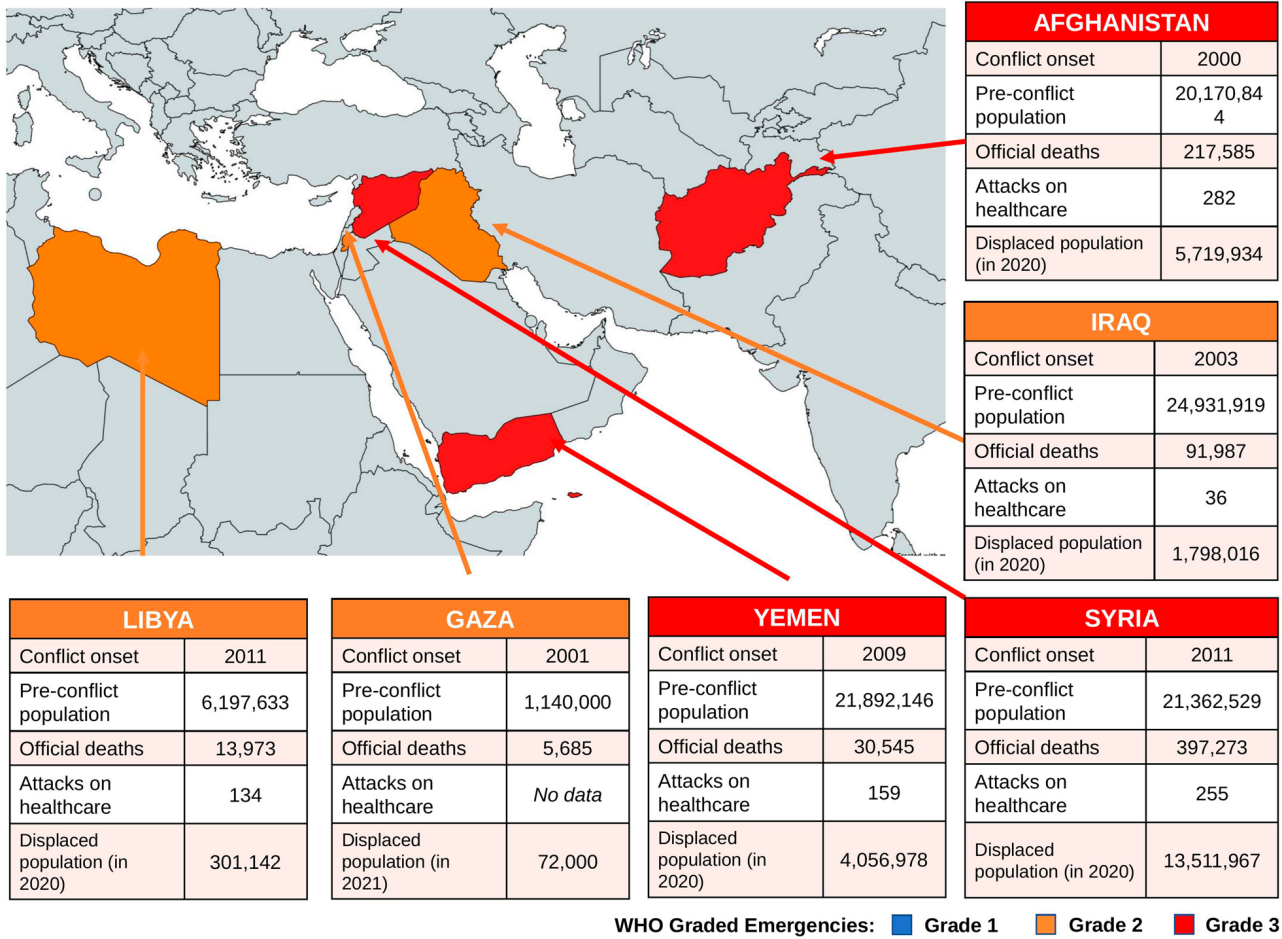
Map of health emergencies in the Eastern Mediterranean Region, as defined by the World Health Organisation’s (WHO) emergency grading, and relevant data on conflicts up to 2020. Grade 1—emergencies with minimal public health consequences, grade 2—emergencies with moderate public health consequences, grade 3—emergencies with severe public health consequences [9]. Conflict onset and number of deaths since onset were sourced from the Uppsala Conflict Data Program [10]. Pre-conflict populations were sourced from The World Bank, and attacks on healthcare sites were from the WHO Surveillance Dashboard [11, 12]. Displaced populations were calculated from the number of refugees, asylum-seekers and internally displaced people for each country, as reported in the UN Refugee Agency, except Gaza where data was taken from UNICEF [6, 13]. Map was created using mapchart.net [14]

Several countries in the World Health Organisation (WHO) Eastern Mediterranean Region (EMR) have been affected by protracted and complex armed conflicts and humanitarian crises [5]. This WHO region oversees 22 countries in Africa, the Middle East and Central Asia and accounts for almost 30 million of the more than 80 million forcibly displaced people worldwide [6, 7]. WHO has classified six countries in the EMR region as health emergencies; these include Iraq, Palestine and Libya at grade 2 and Afghanistan, Yemen and Syria as grade 3 as of 2022 [8]. See Fig. 1.

These countries have faced numerous public health challenges due to conflicts, with most facing deliberate attacks on healthcare facilities and healthcare workers [1]. For example, in Syria, more than 923 healthcare workers have been killed during the conflict and there have been 600 attacks on healthcare facilities in Syria [15]. This has led to the forcible displacement of thousands of Syria's healthcare workers with almost 50% of Syria’s health facilities rendered non-functional leaving a significant gap in healthcare provision [16]. As of 2020, one third of the global deaths caused by conflict occurred in Syria, Afghanistan and Iraq alone [10].

Despite armed conflict causing destruction, these challenging circumstances can be an important catalyst for innovation including the introduction of widespread use of antibiotics and surgical techniques [17]. Some of these have been incorporated into non-conflict healthcare practice. More recently, technological innovations have included 3D limb printing for prosthesis and the use of telemedicine and tele-education initiatives to support the health systems of conflict-affected countries [4, 18].

### Telemedicine

WHO has been a proponent for the use of telemedicine for some years and in 2005 it set up a Global Observatory for eHealth (the use of information and communication technologies for health), of which telemedicine is a part [4]. WHO has advocated the use of eHealth strategies with the aim, among other goals, to support the Sustainable Development Goals on Universal Health Coverage [19]. There are several examples of telemedicine interventions in low- and middle-income countries (LMICs) which include telecardiology, teleradiology, tele-mental health, tele-intensive care (ICU) and teledermatology [20]. Impediments to the integration of telemedicine solutions in LMIC or conflict-affected settings include insufficient resources (financial, material and human), poor connectivity and unreliable electricity supplies [20]. Other factors include the poor evidence base, particularly in conflict-affected settings, poor implementation and lack of evaluation of impact [20].

The aim of this systematic review was to identify the scope and nature of telemedicine interventions in six conflict-affected settings in the EMR which represent different emergency grades on the WHO grading system.

## Methods

We conducted a systematic review of academic and grey literature between January 1st 2000 and December 31st 2020.

### Eligibility criteria

Studies using telemedicine in Libya, Yemen, Gaza, Syria, Iraq and Afghanistan were included. Of the Palestinian territories, only Gaza was included because it faces unique challenges from economic blockade and air strikes [21]. Displaced populations of the included countries who have settled in neighbouring EMR countries were also included. However, interventions for those settled in high income countries and military populations were excluded due to different resources available in these regions. The Uppsala Conflict Data Program was used to identify the date when conflict started in each of these countries and thus, the study period to be included [10]. Studies conducted in a post-conflict era were also included, due to experiencing similar challenges to conflict periods [22]. Only conflicts occurring after 2000 were used since technology before this time may not be relevant for future telemedicine practice [4].

There were various definitions of telemedicine in the literature, however, we adapted the WHO’s definition to: *using information and communication technologies by any healthcare worker, across a distance, for the diagnosis and treatment of disease and injuries* [4, 23]. Non-clinical interventions such as public health measures, patient or healthcare worker education, and improving research opportunities were excluded.

To synthesise all examples of telemedicine in conflict settings, all study designs were included. Eligible outcome measures were effects on patients or healthcare workers, challenges experienced, and requirements for implementation such as technical, logistical, legal and ethical. See Table 1 for a summary of the eligibility criteria.

**Table 1:** Eligibility criteria used to assess study suitability for this systematic review

Criteria	Included	Excluded
Language	English	Non-English
Population	*Populations in receipt of telemedicine and:* Residents of Libya, Yemen, the Gaza Strip, Syria, Iraq, Afghanistan Refugees and undocumented migrants originating from the conflict-affected countries but now residing in neighbouring Middle Eastern countries (including Turkey, Lebanon, Jordan, Israel, Iran, Pakistan)	Military personnel and veterans Refugees originating from conflict-affected countries but now residing outside of the Middle East Telemedicine use for an individual patient only
Intervention	Carried out by healthcare worker Used information and communication technologies For diagnosis and treatment of disease and injury The healthcare worker and receiver must be separated by geographical space—including a healthcare worker and patient, or healthcare worker and another healthcare worker receiving training	Health administration Assessment of feasibility for telemedicine intervention (if preceded telemedicine implementation)
Outcomes	*Primary outcomes:* Patient health outcomes Patient perspectives Healthcare staff perspectives *Secondary outcomes:* Financial Programmatic Ethical Legal Technical	
Study design	Quantitative and qualitative primary research articles Textual (including commentaries, and editorials)	
Time period (from conflict onset)	Libya: February 2011–December 2020 Yemen: January 2009–December 2020 Gaza: January 2001–December 2020 Syria: March 2011–December 2020 Iraq: March 2003–December 2020 Afghanistan: January 2000–December 2020	

### Searches

Ten electronic databases were systematically searched, following the Preferred Reporting Items for Systematic Reviews and Meta-Analysis (PRISMA) guidelines and this was completed in January 2022. MEDLINE, Embase, Global Health, HMIC, MIDIRS, PsychInfo, Web of Science, Scopus, Cochrane Library and CINAHL were searched for articles published from 1st January 2000 to 31st December 2020. Search terms included keywords and subject heading terms that were synonyms of telemedicine, telecommunications and the six conflict settings outlined. For grey literature, googlescholar.co.uk, who.int and msf.org.uk were searched to capture key literature, using 12 search phrases that included ‘telemedicine’ or ‘telehealth’ and the country. See Additional file 1 for detailed search strategies.

Results were imported into Covidence (Covidence.org, Melbourne) for duplicate removal and screening. Two reviewers (PP, RS) systematically screened study titles and abstracts, followed by the full text, using the eligibility criteria. Only studies published in English and with a full text available were included. Discrepancies between reviewers were discussed after each stage of screening and resolved together.

### Data extraction and risk of bias assessment

Extracted data included study design, objectives, setting of both telemedicine provider and recipient, population characteristics, study period, telecommunication type, telemedicine speciality, outcome measures and any other themes within the text. Data extraction was performed by one reviewer (PP). Studies that were the pilot form of another already included study were omitted from data extraction.

The Joanna Briggs Institute checklist for assessing risk of bias was used for case reports and commentaries and used 6 domains [24]. The National Heart, Lung and Blood Institute’s quality assessment tools were used for observational and experimental studies, assessing up to 14 domains [25]. For commentaries, reliability of authors, use of analytical processes and references to relevant literature were examined. The NHLBI assessments covered clarity of objectives, eligibility criteria, participation rate, sample size, outcome measures and statistical analysis as well as study period and use of randomisation where applicable. Each domain for risk of bias was graded as ‘yes’, ‘no’ or “cannot determine” and a score was calculated from the percentage of domains marked ‘yes’. Each study was categorised as low (≤ 49%), moderate (50–74%), or high (≥ 75%) quality.

### Data synthesis

Due to heterogeneity of study design and the scoping nature of the review, a narrative synthesis of the data was performed. Using the Economic and Social Research Council guidance for narrative synthesis, themes were identified from the data, the similarities and differences between data were explored and the strength of evidence was assessed [26].

## Results

**Fig. 2:**
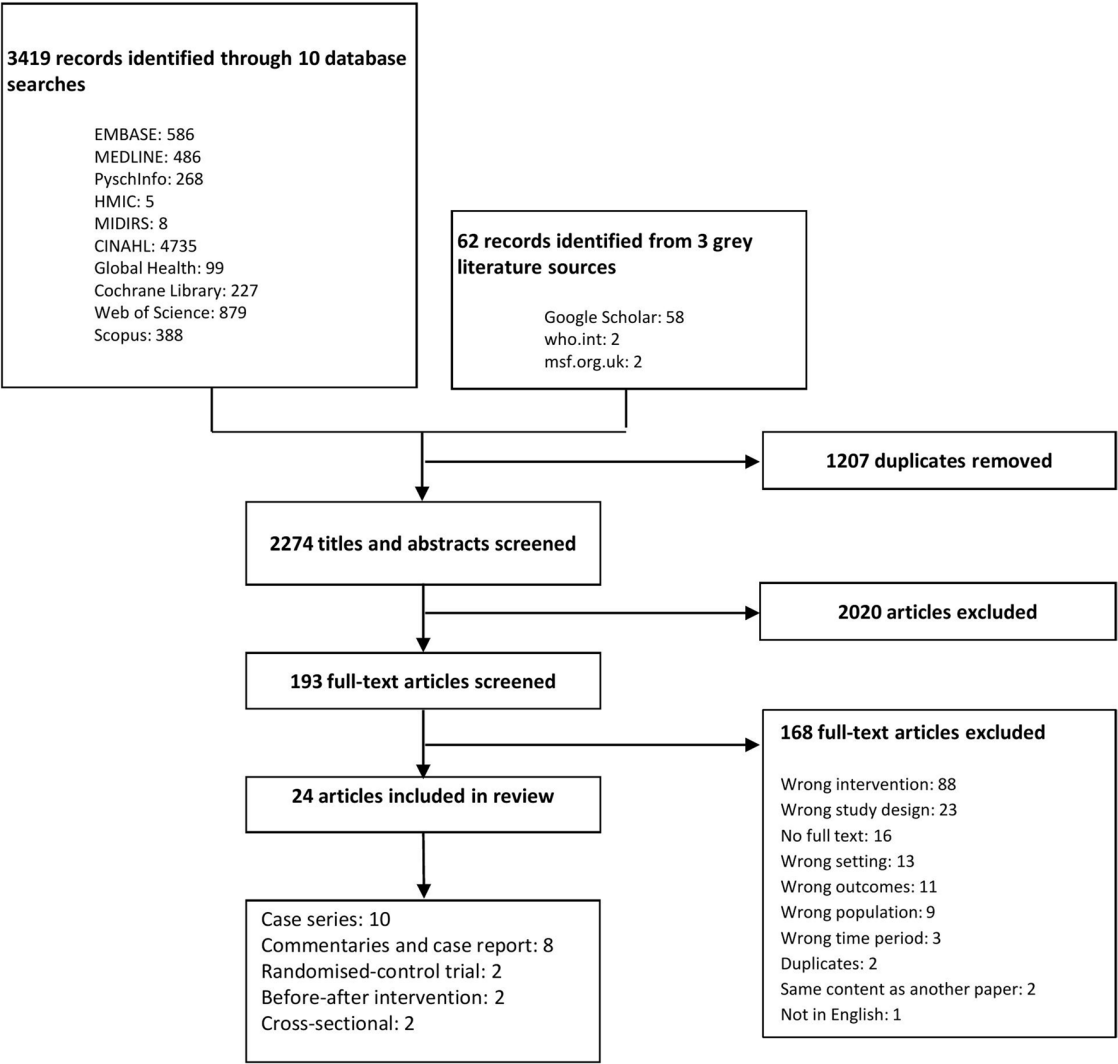
PRISMA flow diagram showing the process of study selection for this systematic review

A total of 3419 articles were identified through database searches and resulting in 193 articles included for full text screening. Of these, 152 studies did not meet the inclusion criteria and 16 full texts were not available in full text despite through Google Scholar. 24 articles were included for analysis. See Fig. 2.

### PRISMA chart (Fig. 2)

**Fig. 3:**
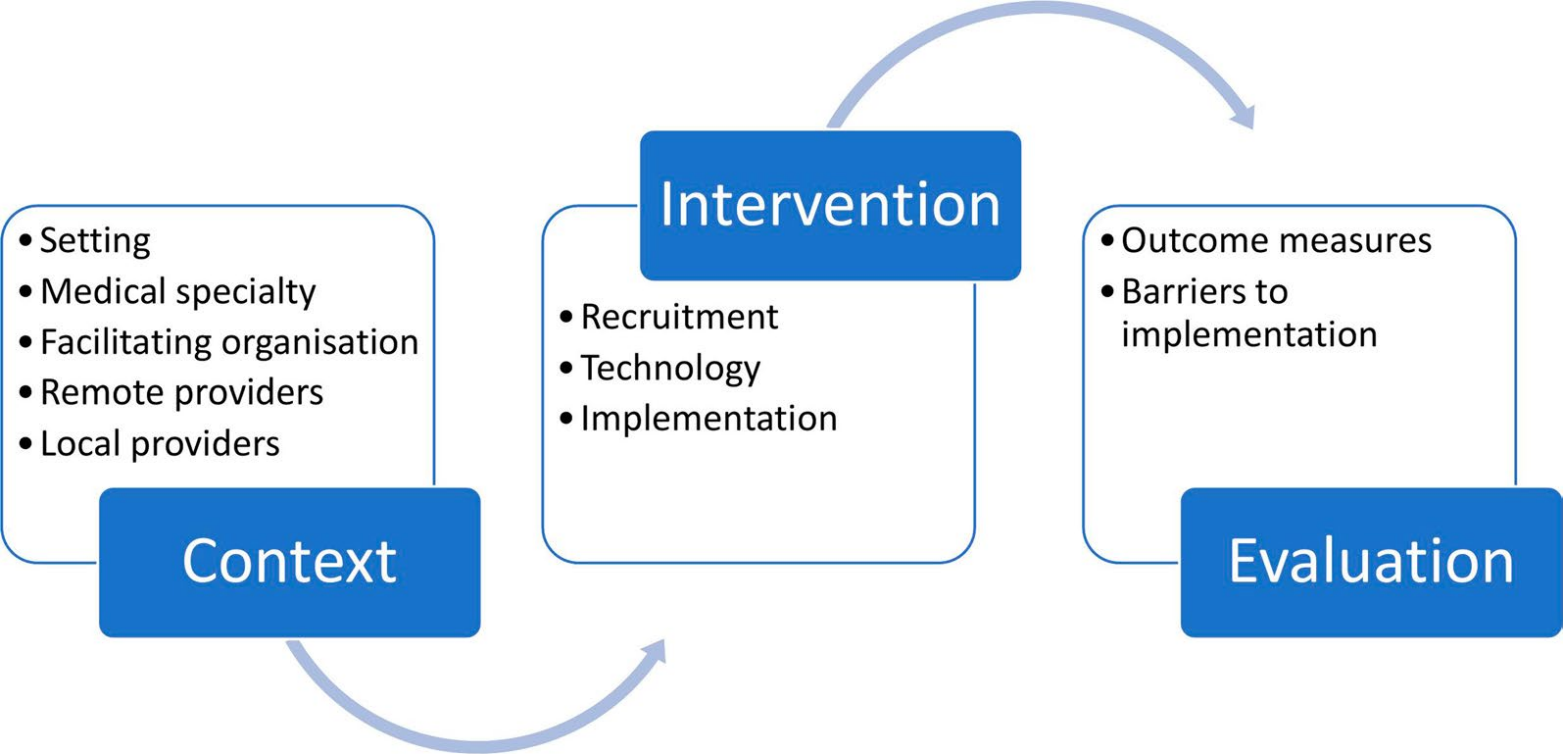
The stages of telemedicine implementation, adapted from Damschoder et al. Consolidated Framework for Implementation Research [51]

Included articles were observational studies (10 articles, all case series) [27–36], commentaries and case reports (8 articles) [37–44], interventional studies (4 articles, randomised control trials and before-after interventions) [45–48] and cross-sectional studies (2 articles) [49, 50] (Fig. 2). Since studies predominantly described implementation rather than effectiveness of interventions, common themes were mapped into a conceptual framework adapted from Damschoder et al. Consolidated Framework for Implementation Research [51]. See Fig. 3.

### The stages of telemedicine implementation in conflict-affected settings (Fig. 3)

#### Context

#### Setting

**Fig. 4:**
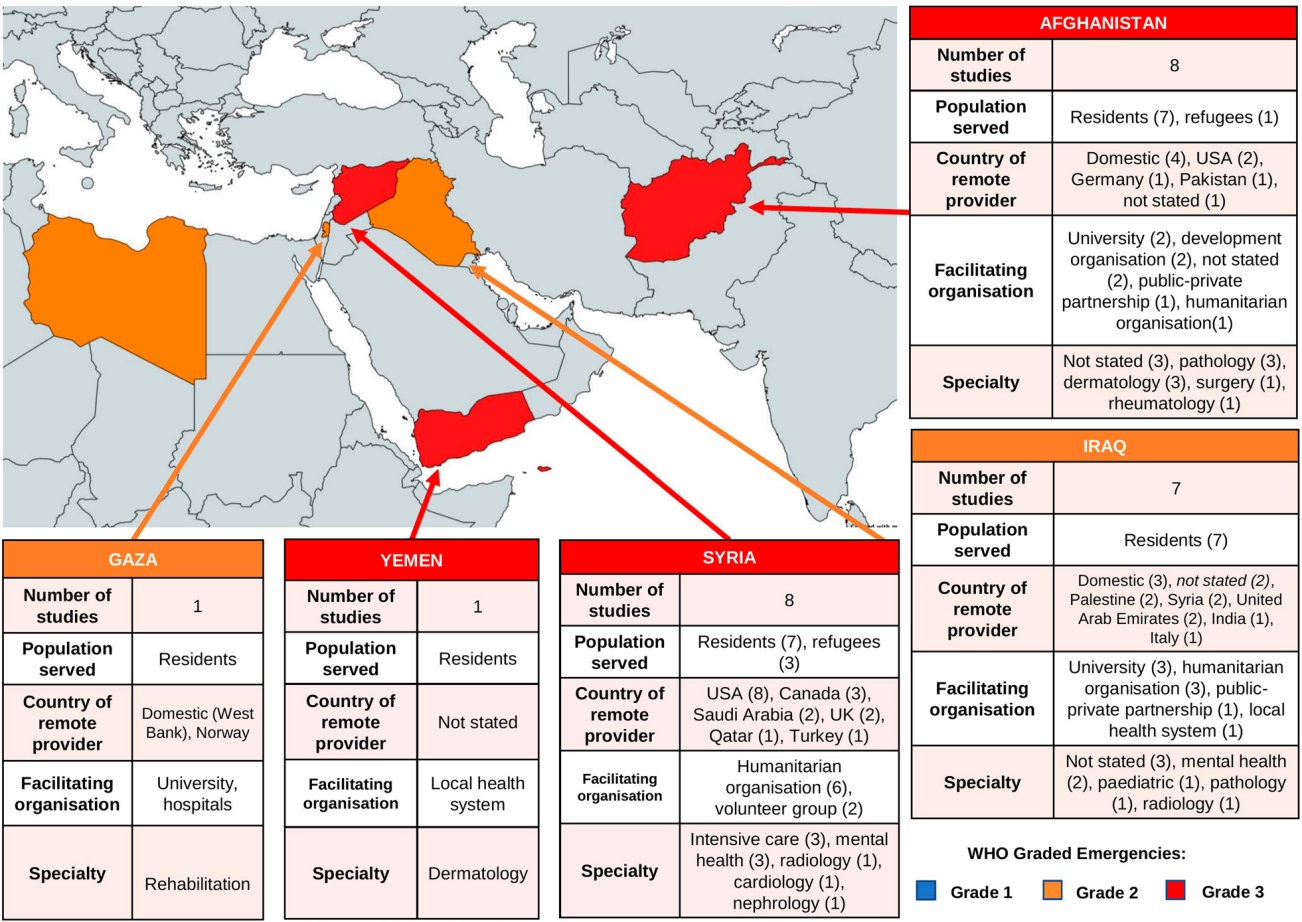
Data on context of all telemedicine interventions, by conflict. Numbers within parentheses indicates number of interventions. *Note*: some interventions covered more than one specialty or country. ‘Population served’ refers to patients treated by telemedicine. ‘Residents’ are residents of the country shown on the map, while ‘refugees’ are settled in another country in the Eastern Mediterranean region. ‘Remote provider’ refers to healthcare workers outside the conflict setting and delivered care through telemedicine

The included studies predominantly described telemedicine interventions for conflict-affected populations from Afghanistan [32, 34–36, 40, 45, 46, 49], Iraq [28, 30, 35, 41, 47, 48, 50] and Syria [27, 29, 31, 33, 38, 39, 43, 44]. There was one study from Yemen [37] and Gaza [42] each, and none from Libya. See Table 2. Most studies were conducted within the conflict setting, except four studies which included refugees in neighbouring countries. See Fig. 4. These were three tele-mental health interventions for displaced Syrians in Lebanon, Jordan, and Tunisia [27, 33, 43], and a tele-rheumatology project for Afghan refugees in Iran [36].

**Table 2:** Characteristics of included articles, ordered by location of intervention (country of patients)

Author and publication year	Study design	Study period	Facilitating organisation	Country of remote provider(s)	Country of patients	Setting of patients	Number of patients treated	Medical specialty	Outcome measures
*Afghanistan*									
Patterson et al. [35]	Case series	2004–2007	Humanitarian organisation (*Swinfen Charitable Trust, UK*)	Multiple (specifics not stated)	Iraq, Afghanistan, Kuwait, Pakistan	Not stated	203 (Iraq), 55 (Afghanistan)	Various	Number of cases, qualitative opinions of referring doctors (2), image attachments included with each case, reply time of remote HCW, number of email exchanges, HCW confidence in diagnosis
Sajwani et al. ^a^ [40]	Commentary	2013–2017	Development organisation (*Government of Canada, Aga Khan Foundation Canada*)	Afghanistan	Afghanistan	Hospital (secondary care)	Not stated	Various	None
Khoja et al. [34]	Case series	2007–2014	Private organisation (*Roshan telecommunications, part owned by Aga Khan Fund for Economic Development*)	Afghanistan, Pakistan	Afghanistan	Not stated	15,000	Radiology, Pathology, Surgery	Number of cases, approximate cost and time savings to patients
Ismail et al. [46]	Before-after intervention	2013–2017	University (*Emory University, USA*)	USA	Afghanistan	Hospital (tertiary care)	150	Dermatopathology	Number of cases, % change of diagnoses
Ismail et al. [32]	Case series	2013–2017	Not stated	Afghanistan	Afghanistan	Clinic (primary care)	326	Dermatology	Number of cases, % cases diagnosis given, % cases local HCW asked follow-up questions
Fritz et al. [45]	Before-after intervention	Not stated	Not stated	Germany	Afghanistan, Tanzania	Not stated	156 (Afghanistan), 10 (Tanzania)	Pathology	Number of cases, % change of diagnoses
Sayani et al. ^a^ [49]	Cross-sectional, retrospective	2013–2017	Development organisation (*Aga Khan Foundation Canada, Global Affairs Canada*)	Afghanistan	Afghanistan	Not stated	19,157	Various	Number of cases, projected time and cost savings to patient
Rezaian et al. [36]	Case series	5 years (date not stated)	University (*West Virginia University, USA*)	USA	Iran (Afghan refugees)	Hospital clinic (outpatients, refugee population)	4800	Rheumatology	Number of cases, number of diagnostic tests performed, number of medications given
*Iraq*									
Swinfen et al. ^b^ [41]	Commentary	2004—2005	Humanitarian organisation (*Swinfen Charitable Trust, UK*)	Not stated	Iraq	Not stated	150	Various	None
Patterson et al. ^b,e^ [35]	Case series	2004–2007	Humanitarian organisation (*Swinfen Charitable Trust, UK*)	Multiple (specifics not stated)	Iraq, Afghanistan, Kuwait, Pakistan	Not stated	203 (Iraq), 55 (Afghanistan)	Various	Number of cases, number image attachments per case, reply time of remote HCW, number of email exchanges, HCW confidence in diagnosis
Al-Hadad et al. [28]	Case series	2000–2009	University (*Sapienza University, Italy*)*,* Humanitarian organisation (*INTERSOS, Italy*)	Italy	Iraq	Hospital	10 per month	Paediatric oncology, Pathology	Number of cases, % change in diagnosis, mortality
Wagner et al. ^c^ [47]	Randomised-control trial	2009–2011	University (*Freie University, Germany*)	Iraq, Palestine, Syria, United Arab Emirates, Europe	Iraq	Patient's home	47	Mental health	Working Alliance Inventory
Knaevelsrud et al. ^c^ [48]	Randomised-control trial	2009- 2011	University (*Freie University, Germany*)	Iraq, Palestine, Syria, United Arab Emirates, Europe	Iraq	Patient's home	159	Mental health	Number of cases, Post-traumatic Diagnostic Scale score, Hopkins Symptom Checklist-25 score, Symptom Checklist-90-Revised score, EUROHIS-QOL score
AbdGhani et al. [50]	Cross-sectional, retrospective	Not stated	Local health system	Iraq	Iraq	Not stated	30	Various	Method of tele-consultation, % of consultations conducted electronically, number of years ago that tele-consulting was started
Belman et al. [30]	Case series	2009–2010	Public–private sector partnership (*CARE Foundation*)	India	Iraq, India, Nigeria	Not stated	2274	Radiology	Number of cases
*Syria*									
Jefee-Bahloul et al. [33]	Case series	2013	Humanitarian organisation (*Syrian American Medical Society, USA*)	USA	Jordan (Syrian refugees)	Refugee camp clinic	6	Mental health	Number of cases
Al-Makki et al. [44]	Commentary	2014–2017	Humanitarian organisation (*Syrian National Kidney Foundation, USA*)	USA	Syria	Hospital	Not stated	Nephrology	None
Jefee-Bahloul et al. ^d^ [43]	Case report	2014—not stated	Humanitarian organisation (*Syrian TeleMental Health Network*)	USA, Canada, UK, Middle East	Syria, Lebanon, Turkey, Jordan (Syrian refugees)	Clinic (primary care)	Not stated	Mental health	None
Moughrabieh et al. [39]	Commentary	2012–2015	Volunteers (*funded by Syrian American Medical Society, USA*)	USA, Canada	Syria	Hospital	90 per month	Intensive care	Number of cases per month, disease presentation type
Alrifai et al. [38]	Commentary	2014—not stated	Humanitarian organisation (*Syrian American Medical Society, USA*)	USA	Syria	Hospital	Not stated	Cardiology, Intensive care	None
Masrani et al. [29]	Case series	2015–2018	Humanitarian organisation (*Teleradiology Relief Group established by Syrian American Medical Society, USA*)	USA, Saudi Arabia	Syria	Not stated	Not stated	Radiology	Number of radiological images interpreted
Ghbeis et al. [31]	Case series	2013–2014	Volunteers	USA	Syria	Hospital	19	Intensive care (paediatric)	Number of cases, consultation recommendations, patient mortality
Almoshmosh et al. ^d^ [27]	Case series	2014–2017	Humanitarian organisation (*Syrian Tele-Mental Health Network*)	UK, USA, Canada, Qatar, Saudi Arabia, Turkey	Syria, Turkey, Lebanon (Syrian refugees)	Not stated	123	Mental health	Number of cases, referral location, type of questions from referring HCWs, type of advice given by provider HCW
*Gaza*									
Olsen et al. [42]	Project report	2006–2009	Hospitals (*Patient Friends Society K. Abu Raya Rehabilitation Centre, Bethlehem Arab Society for Rehabilitation, Jerusalem Princess Basma Centre for Disabled Children, El Wafa Medical Rehabilitation Hospital, Sunnaas Rehabilitation Hospital*), WHO collaborating centre (*Norwegian Centre for Integrated Care and Telemedicine*), Development organisation (*Norwegian Association of Disabled*), Private organisation (*Tanberg*)	West Bank and multiple international partners	Gaza	Hospital (rehabilitation specialist hospital)	Not stated	Rehabilitation	None
*Yemen*									
Al-Kamel et al. [37]	Commentary	2013—not stated	Local health system (*Regional Leishmaniasis Control Center*)	Not stated	Yemen	Not stated	Not stated	Dermatology	None

‘Facilitating organisation’ relates to organisation that implemented the intervention. ‘Humanitarian organisation’ includes charities, non-governmental organisations and non-profit organisations. ‘Remote providers’ relates to healthcare workers at a different setting to their patients and delivered healthcare through telemedicine

HCW, healthcare worker

^abcd^Each pair of articles report on the same intervention

^e^Patterson et al. [35] appears twice within the table due to operating in both Afghanistan and Iraq

Four groups of stakeholders were involved in the telemedicine interventions: facilitators, remote providers, local providers and patients. Facilitators coordinated logistics and sometimes funded interventions; remote providers were healthcare workers outside of conflict settings, while local providers worked within the conflict and would provide direct care to patients. Where stated, different healthcare settings were represented in the studies. Most interventions were in hospitals [28, 31, 36, 41, 42, 44, 46, 49, 50], and urban areas [28, 34, 36, 41, 42, 46, 49, 50]. However, four studies were conducted in primary care facilities [32, 33, 43, 49] and three studies specifically aimed to reach rural participants in Afghanistan [34, 49] and Yemen [37]. Most interventions created a network of remote and local providers across numerous clinical sites [27, 30–32, 34, 35, 37–40, 42–45, 48–50], while 3 studies involved communication between just two sites [28, 33, 36].

#### Medical specialty

Telemedicine was represented in ten specialities across conflict settings (Fig. 4) and aimed to alleviate the shortage of specialist healthcare workers. Broadly, three types of telemedicine were used:

- Exchange of images for interpretation and diagnosis
- Exchange of a clinical case for advice on diagnosis or treatment
- Direct interaction of remote provider with a patient

Specialties only exchanging images and short text accounted for four studies, including transfer of histological slides in tele-pathology [45] and tele-dermatopathology [46] interventions from Afghanistan. Additionally, Digital Imaging and Communications in Medicine (DICOM) transmission of radiological images in tele-radiology interventions from Iraq [30] and Syria [29]. This was done asynchronously with Store and Forward (SAF) technology.

All other specialties sent a written clinical case and question to a remote provider, although some also attached images and audio-visual files. Overall, interventions varied across specialties and conflict settings. In Afghanistan, two email-based interventions sent case histories for advice on diagnosis of dermatological conditions [32], and management of complicated cases across multiple specialties including obstetrics and neurology [35]. Meanwhile in a tele-rheumatology intervention, a remote provider conducted video-consultations with patients and a local provider [36]. Two other interventions that covered multiple specialities, including surgery and radiology, also utilised videoconferencing to discuss cases [34, 49].

In Iraq, a tele-paediatric intervention asked remote providers to discuss difficult cases and update treatment guidelines to match international standards [28], while two tele-mental health interventions guided patients through therapy using structured writing assignments [47, 48].

In Syria, interventions were conducted in intensive care (ICU) [31, 39], cardiology [38], radiology [29], nephrology[44] and mental health [27, 33, 43]. In both tele-ICU and tele-cardiology, remote providers triaged, interpreted test results and created management plans for acutely ill patients in real-time using instant messaging and video-calls [31, 38, 39]. In one tele-mental health intervention, a remote provider video-called a single local provider to discuss treatment-resistant patients [33], while other mental-health interventions involved multiple local providers submitting cases to a referral system for review by a remote provider [27, 43].

In Yemen, a tele-dermatology intervention used social media communication for follow-up appointments between local providers and rural patients, while remote providers were contacted for advice [37]. In Gaza, a tele-rehabilitation intervention set-up videoconferencing between local rehabilitation hospitals to support each other [42].

#### Facilitators

Interventions were mostly facilitated by humanitarian, academic and development organisations based outside of the conflict setting, predominantly from the United States of America (USA) and Europe (Fig. 4). All eight interventions for Syrians were facilitated by USA-based humanitarian organisations, including three projects by the Syrian American Medical Society (SAMS) [29, 33, 38]. Of eight interventions in Afghanistan, two were coordinated by USA universities [36, 46], and two were implemented by the Pakistan-based Aga Khan Development Network [40, 49], and one by a private telecommunications company [34]. Of seven studies from Iraq, facilitating organisations were varied. Three studies came from universities in Germany and Italy [28, 47, 48], two from a UK-based humanitarian organisation (the Swinfen Charitable Trust) [35, 41], one by an Indian public–private partnership [30], and one was locally coordinated in Baghdad [50]. The studies from Yemen and Gaza were partly facilitated locally but received assistance from international partners. The Palestinian rehabilitation hospitals approached the Norwegian Centre for Integrated Care and Telemedicine [42], while the Regional Leishmaniasis Control Centre in Yemen did not state its collaborators [37].

#### Intervention

#### Recruitment of remote providers

Remote providers were mostly recruited by the facilitating organisation and based outside of the conflict setting (Fig. 4), apart from four studies in Afghanistan [32, 34, 40, 49] and three in Iraq [47, 48, 50] where local healthcare workers participated. Some studies recruited providers based on skillset and level of commitment [31, 35, 39], while all five tele-mental health and one tele-ICU studies recruited only Arabic-speaking providers for improved communication with local healthcare workers and patients [27, 33, 39, 43, 47, 48]. Most studies enlisted specialist doctors [27, 29–31, 33–36, 38–40, 43–46, 50], while three utilised psychologists [27, 47, 48], one recruited biomedical engineers [44] while five did not state profession [28, 32, 37, 41, 42]. Five studies also stated that their remote providers were volunteers [27, 29, 31, 35, 39], and these studies were facilitated by humanitarian organisations. The remaining studies did not detail renumeration of providers.

#### Recruitment of local providers

When recruiting local providers, some telemedicine facilitators identified suitable providers by enlisting local partners [27, 28, 33, 38, 39, 46], while in other studies, local providers approached facilitators instead [35, 40, 41, 45]. Four Syria-based studies gained local partners through other humanitarian activities of the facilitators [29, 31, 33, 38], and two studies utilised academic collaborations with universities in Iraq and Afghanistan [28, 46]. To determine suitability of the setting for telemedicine and successful implementation, four studies conducted needs assessments to evaluate limitations of resources available, such as staffing and medical equipment[27, 33, 39, 49]. This also guided which resources were supplied during preparation for the intervention [39, 43, 49]. Another three studies identified a strong commitment towards the intervention from the medical director as an important factor for successful implementation [31, 40, 43].

#### Technology

Two types of technology were featured in the included studies; 14 studies used electronic referral systems [27, 30, 32, 35–37, 39, 41, 43, 45–50] and 8 studies used social media applications [29, 31, 33, 36–39, 44] (Table 3).

**Table 3:** Technology used by telemedicine interventions to enable communication between remote providers, local providers and patients

Technology	Conflict setting	Author	Specialty	Transmission type
*Electronic referral system*				
iPath	Afghanistan	Fritz et al. [45]	Pathology	SAF
Email	Afghanistan	Ismail et al. [46]	Dermatopathology	SAF
Email	Afghanistan	Ismail et al. [32]	Dermatology	SAF
Electronic medical records database	Iran (Afghan refugees)	Rezaian et al. [36]	Rheumatology	SAF
Virtualdoc	Afghanistan	Sayani et al. [49]	Various	SAF
Email (via AutoRouter)	Afghanistan and Iraq	Patterson et al. [35]	Various	SAF
Email	Iraq	Swinfen et al. [41]	Various	SAF
Email	Iraq	Belman et al. [30]	Radiology	SAF
Iterapy	Iraq	Knaevelsrud et al. [48]	Mental health	SAF
Iterapy	Iraq	Wagner et al. [47]	Mental health	SAF
Email	Iraq	AbdGhani et al. [50]	Not stated	SAF
Collegium Telemedicus	Syria	Jefee-Bahloul et al. [43]	Mental health	SAF
Horos, OsiriX Lite	Syria	Moughrabieh et al. [39]	Intensive care	SAF
Collegium Telemedicus	Syria	Almoshmosh et al. [27]	Mental health	SAF
Email	Yemen	Al-Kamel et al. [37]	Dermatology	SAF
*Social media application*				
Skype	Syria	Al-Makki et al. [44]	Nephrology	RT
Facebook, WhatsApp, Viber, Google Hangout	Syria	Alrifai et al. [38]	Cardiology	SAF + RT
Facebook, WhatsApp, Telegram	Syria	Masrani et al. [29]	Radiology	SAF
Facebook, Skype	Syria	Ghbeis et al. [31]	Intensive care	SAF + RT
Skype	Syria	Jefee-Bahloul et al. [33]	Mental health	RT
Skype, WhatsApp, Viber, Google Hangout	Syria	Moughrabieh et al. [39]	Intensive care	SAF + RT
Facebook, WhatsApp, SMS, phone calling	Yemen	Al-Kamel et al. [37]	Dermatology	SAF + RT
Skype, telegram	Iran (Afghan refugees)	Rezaian et al. [36]	Rheumatology	SAF + RT

SAF, store and forward; RT, real-time

Electronic referral systems enabled SAF (Store and Forward) referral of case notes, images and questions, which could then be viewed and answered by remote providers in their own time. Most studies using electronic referral systems were based in Iraq [30, 35, 41, 47, 48, 50] and Afghanistan [32, 35, 36, 45, 46, 49], and conducted tele-mental health, tele-dermatology and tele-pathology interventions as well as studies working across multiple specialties (Table 3).

The electronic referral systems were either email-based systems [30, 32, 35, 37, 41, 46, 50], or a specialised platform [36, 39, 43, 45, 47–49] such as Collegium Telemedicus [27, 43]. Where stated, laptops and computers were used to access these platforms, and some required specialised software, such as integration with DICOM for tele-radiology [30]. Cameras were also required in a tele-mental health intervention to record patient consultations [43], and a tele-pathology intervention used software to enabled discussion forums for specialists to discuss cases [45]. Some systems had additional functions such as allocating cases to a network of remote providers based on clinical urgency (tele-radiology) [30], or to providers of appropriate specialty and availability, where the intervention spanned multiple specialties [35]. In tele-mental health [43], tele-ICU [39], and tele-rheumatology [36], referral systems stored health records which improved efficiency and enabled data encryption.

Social media applications enabled both SAF and real-time transfer of text, images, audio and videos for communication between providers. Six out of eight studies were from Syria [29, 31, 33, 38, 39, 44] and utilised in several specialties (Table 3). The most common applications were WhatsApp, Facebook Messenger and Skype. Reasons to use social media included low cost [38], functionality with low internet bandwidth [31], minimal training prior to use [39], and easy access to hardware such as mobile phones and laptops [37, 38].

Functionality with accessible equipment such as mobile phones enabled providers in Syria to photograph hard-copy radiograph films and record ultrasound scans where radiology equipment did not support electronic transfer of images [29]. Instant messaging, such as WhatsApp, was compatible with limited internet bandwidth where videoconferencing would not have been possible [31] and this allowed almost real-time information sharing for 24-h monitoring of ICU patients [31, 39]. Audio messages were even more convenient for remote providers in tele-ICU since they require less time to record than text [31]. Additionally, real-time telemedicine was possible using Skype and Viber so remote and local providers could discuss cases in tele-mental health, tele-rheumatology and tele-nephrology interventions [33, 36, 44]. Both SAF and real-time technology were used by tele-rheumatology and tele-ICU interventions since they used social media for communication between providers, and an electronic medical records platform to store patient information.

#### Evaluation

#### Outcome measures

Outcome measures were reported in 17 of the 22 included studies [27–36, 39, 45–50], although they were heterogenous and mostly without statistical analysis. Outcome types were categorised into patient-related and provider-related, and seven studies came from Afghanistan [32, 34–36, 45, 46, 49], six from Iraq [28, 30, 35, 47, 48, 50], and five from Syria [27, 29, 31, 33, 39].

Sixteen studies reported patient-related outcome measures, of which, 14 studies measured the number of cases treated by telemedicine[27, 28, 30–32, 34–36, 39, 43, 45, 46, 48, 49]. Mortality rates were reported in two tele-paediatric interventions [28, 31], while two interventions that involved multiple specialties predicted the cost and time savings to patients [34, 49]. A tele-ICU study determined the proportion of patients that needed treatment for traumatic injuries [39], a tele-rheumatology study recorded rheumatological diseases [36], while a tele-mental health study used psychiatric diagnostic scales to measure post-traumatic stress symptoms, depression, somatisation and quality of life [48].

Nine studies reported on provider-related outcomes including four studies that reported on the percentage of diagnoses that changed following tele-pathology [45, 46], tele-dermatology [32] and tele-paediatric consultations [28]. The types of clinical advice given by the remote provider were measured in a tele-ICU intervention including frequency of drug prescriptions, resuscitation and ventilation instructions being given and seizure management [39]. In a tele-rheumatology study, frequency of drug prescriptions and radiological and serological tests was measured [36].

#### Barriers to implementation

Barriers to implementation included factors related to facilitators, providers, and technology, and were reported in 16 studies [27, 29–33, 35–37, 39–42, 46, 49, 50], across all conflict settings and medical specialties. The single most common barrier concerned technology, specifically limited internet bandwidth, and was reported in 12 studies [29–31, 33, 36, 37, 39–42, 46, 49]. For example, internet was unreliable which led to interruption of synchronous video-calls in tele-mental health [33] and tele-rheumatology [36] interventions, although audio calls suffered less. Low bandwidth also led to slow image transfer in tele-radiology [29, 30] and tele-ICU [39], and poorer quality images in a tele-dermatology study [46]. Of these studies, six used electronic referral systems [30, 40, 42, 46, 48, 49] and six used social media [29, 31, 33, 36, 37, 39]. Additionally, six studies also had difficulties acquiring adequate technical equipment [29, 36, 37, 42, 46, 49]. This particularly affected interventions aiming to capture high quality images in tele-dermatology [37], dermatopathology (45), rheumatology [36] and radiology [29]. Two interventions from Afghanistan cited expense as a barrier [46, 49], while a study from Gaza faced import restrictions due to economic blockade [42].

Of all barriers reported, most were provider related and concerned both remote and local healthcare workers. Seven studies described limited availability of healthcare resources, specifically staff shortages in three studies, including two tele-ICU [31, 39] and a tele-rheumatology [36] intervention. Conflict also caused damage to healthcare facilities in interventions from Syria [27, 39] and Iraq [28]. Meanwhile, tele-ICU [39], tele-radiology [29], tele-dermatopathology [46] and tele-mental health [27] interventions reported not having medical supplies such as medications, ventilation and monitoring equipment, computerised tomography (CT) contrast and laboratory testing reagents.

Facilitator related barriers concerned funding limitations and inability to evaluate the interventions for future development. Across all types of facilitating organisations, limited funding affected the sustainability of interventions [31, 33, 37, 39, 40, 42, 46]. The reliance on volunteers affected the continuity of the projects; this was particularly so for tele-ICU studies in Syria [31, 39] since mass casualties and intensive care monitoring required long hours of supervision by remote providers.

### Quality of included studies

Using quality appraisal checklists, 9 articles were graded as low quality [28, 30, 33, 34, 40, 46–48, 50], 7 were moderate quality [29, 31, 36, 41, 42, 45, 49] and 8 were high quality [27, 32, 35, 37–39, 43, 44] (see Additional file 2). The high quality studies were limited to commentaries and case series. Broadly, studies were deemed low quality because of unclear inclusion criteria, lack of blinding of outcome to assessors and lack of statistical analysis.

## Discussion

This systematic review highlights the range of telemedicine interventions in five diverse, conflict-affected settings in the EMR (noting the absence of any in Libya). It also identifies some of the challenges faced in establishing and sustaining such projects. During the COVID-19 pandemic, the use of telemedicine has increased and the need for low-cost, sustainable interventions has become even more pertinent both in these settings and in high income countries (HICs) [52]. We note the use of telemedicine across a range of specialties including pathology, intensive care, dermatology, nephrology and mental health and different models of delivering telemedicine interventions. Key enablers were foreign charitable and academic organisations that coordinated the interventions, and simple telecommunications systems such as social media and electronic SAF platforms. Barriers to implementation concerned health and technology infrastructure, financial limitations, reliance on volunteers and sustainable funding. It is notable that few interventions fully explored pertinent concerns around data storage, confidentiality and ethical standards and what effects these had on local healthcare worker or patient acceptability.

In general, literature on telemedicine in LMICs has been limited in scope, study design and quality with particular gaps in the evaluation of impact and cost effectiveness of such interventions [53]. In HICs before the COVID-19 pandemic, telemedicine projects were mainly focused on the provision of care to rural populations [54]. The growth of telemedicine projects in LMICs has often focused on informal or small-scale interventions which have also supported capacity building through tele-education and research collaborations [4]. A literature review of tele-mental health interventions in post-disaster settings in the Middle East note that telemedicine can bring care to disadvantaged populations though challenges to implementation included patient acceptance, insufficient technology, poor health infrastructure, and political instability [55].

### Externally led initiatives

Most interventions described in this review were initiated by external organisations, whether academic or humanitarian and were based in the USA or Europe; members of such organisations often had a personal link to the setting in which the project was implemented. This structure has not been fully explored in the wider literature on telemedicine in post-conflict settings in terms of the pros and cons of this model [56, 57]. These organisations often filled a gap that the local health system or government could not or was unwilling to fill due to limitations cited as high infrastructure costs, insufficient technical knowledge, and a perceived lack of demand [4]. The projects implemented aimed to tackle such barriers and were able to use either modest charitable or foreign development funding to provide simple telemedicine interventions. However, insufficient funding was often cited as a barrier to sustainability of such programs. Despite this limitation, few projects demonstrated the governance mechanisms or the study design which would be required to demonstrate efficient and effective use of funds to greatest clinical benefit [58].

### Extrapolating innovations

Drawing from the wider literature, we suggest that telemedicine in resource constrained settings could provide an opportunity for reverse-innovation as provider healthcare workers can learn from clinical and technological adaptions in low resources settings [59]. Interventions in conflict settings where funding is scarce could be a catalyst for innovation. In Syria, where ongoing conflict has strained resources, the tele-ICU was set up using low-cost equipment such as webcams, mobile phone cameras and instant communication through free social media applications [38, 39]. This supports literature suggesting that telemedicine could be versatile across medical specialties and settings, particularly with the advent of widespread mobile phone coverage globally [19, 60]. The impetus for this initiative were the dire needs in Syria, particularly in besieged areas where easy-to-source equipment that required minimal training for use was the most practicable [39]. Since mobile phone and social media use is widespread even in conflict settings, and mobile telemedicine is increasingly available globally, this is often used. Though there is concern regarding interception of messages, ethical standards and confidentiality in such settings of extreme conflict, these interventions can be lifesaving [19, 61].

### Investing in healthcare workers

It has been suggested in the literature that poor digital literacy or little prior knowledge of telemedicine may lead to reluctance to adopt a telemedicine program [62]. However, this is likely to be changing during the COVID-19 pandemic. Gaps in knowledge or skill provide an opportunity for capacity building through training which can have long term impacts in the local workforce [4, 63]. This requires investment of time by the provider, developing partnerships and trust and empowering local staff champions [56]. With time and training, this could upgrade local skills and potentially reduce reliance on remote health professionals except for the most complex cases [64]. As such, tele-education forms an integral part to any telemedicine program [4].

### Strengths and limitations

A strength of this study is that we explore the different types and models of telemedicine interventions in select countries in the EMR in both academic and grey literature and note what innovations and gaps exist. This is timely as the COVID-19 pandemic has increased the use of telemedicine interventions globally including in conflict-affected settings. Limitations include the sparsity of literature, much of which was descriptive and of low quality. This may limit the generalisability of the findings particularly as there is large intra and inter-country variation with regards to the availability of resources, a reliable internet connection and trained personnel. In addition, we did not explore other eHealth interventions (i.e., tele-education, electronic health records, and self-help mobile applications) as they are beyond the focus of this study though they have potentially important public health impacts [65]. We only reviewed studies in English which may have missed some interventions, however most published academic studies are likely to be in English rather than Arabic or Pashtun.

## Conclusion

Telemedicine interventions are feasible and needed in conflict settings in the EMR, particularly during and after the COVID-19 pandemic. Though the literature presents a range of different telemedicine interventions with varied models of care, few explore the ethical considerations, governance aspects, clinical outcome evaluation and sustainability of the interventions. There is a demonstrated need for localised interventions appropriate to the setting and the needs of the local health professionals and populations. Evaluation methods and therefore study designs may need to be tailored to LMICs to acknowledge population needs, local institutions capacity and readiness, and the cultural, environmental, economic, legal and policy factors. Experiences of telemedicine interventions in conflict-affected settings in the EMR could inform stakeholders (including medical associations, humanitarian organisations, public health bodies) aiming to provide support to conflict-affected and low-resource settings. These aspects require further exploration with a focus on patient experience and clinical outcomes.

## Academic database and grey literature search strategy

### Academic database search

A search strategy was generated and duplicated for all academic databases and only modified where MESH terms differed. The following databases were searched with the OVID search engine: EMBASE, MEDLINE, MIDIRS, PsychInfo, Global Health, HMIC, CINAHL. In addition, the following databases were searched individually: Scopus, Web of Science, Cochrane Library. See Table 4 for example of search strategy used for EMBASE database.

**Table 4:** Example search strategy used to search EMBASE database

Search strategy	EMBASE Searched on 05/03/2020 -> 522 results
1	(Iraq/ OR iraq* OR baghdad OR erbil OR basrah OR basra OR Syrian Arab Republic/ OR syria* OR aleppo OR damascus OR idlib OR Afghanistan/ OR afghan* OR kabul OR helmand OR Yemen/ OR yemen* OR sanaa OR sana’a OR Libyan Arab Jamahiriya/ OR libya* OR tripoli OR benghazi OR gaza* OR “gaza strip”)
2	(telemedicine/ OR exp telehealth/ OR tele* OR telenursing/ OR ehealth OR "e-health" OR "emental health" Or "e-mental health" OR "emedic*" OR "e-medic*" OR econsult* OR "e-consult*" OR ediagnos* OR "e-diagnos*" OR "video conferenc*" Or "mobile health" OR mhealth OR "m-health" OR videoconferencing/ OR videoconferenc* OR information technology/ OR information communication tech* OR information tech* OR (store adj2 forward) OR (Mobile adj2 tech*) OR Elearning OR "e-learning")
3	((internet or digital* or "web-based" or provider or mobile or online or smartphone* or cell*phone* or telephone* or "mobile phone*" or phone* or "phone based" or "text-messag*") adj2 (treat* or intervention* or therap* or consult* or medic* or prescri* or diagnos* or care or manag*))
4	((mobile or distance) adj2 (educat* or training or learning))
5	2 OR 3 OR 4
6	1 AND 5
7	Limit to yr = “2000–2020”

### Grey literature search

An exhaustive list of terms was used for grey literature search: telemedicine Syria, telemedicine Yemen, telemedicine Libya, telemedicine Iraq, telemedicine Afghanistan, telemedicine Gaza, telehealth Syria, telehealth Yemen, telehealth Libya, telehealth Iraq, telehealth Afghanistan, telehealth Gaza.

Each phrase was searched individually in Google Scholar (scholar.google.com), World Health Organisation website (who.int) and Medecins Sans Frontiers website (msf.org.uk).

## Supplementary Information

**Additional file 1**. Academic database and grey literature search strategy. Full search strategy used for both academic database and grey literature search.

**Additional file 2**. Quality assessment of included studies. The method of quality appraisal of included studies is illustrated by the checklists used to assess quality and risk of bias, as well as data used to generate a quality score for each study.

## Data Availability

The search strategy and quality assessment of included articles is available in additional files. Extracted data are available from corresponding author on reasonable request.

## Abbreviations

WHO: World Health Organisation
EMR: Eastern Mediterranean Region
UNICEF: United Nations International Children's Emergency Fund
LMIC: Low- and middle-income countries
ICU: Intensive Care Unit
COVID-19: Coronavirus disease of 2019
PRISMA: Preferred reporting items for systematic reviews and meta-analysis
DICOM: Digital imaging and communications in medicine
SAF: Store and forward
CT: Computerized tomography
HIC: High income countries
