# Supplementary material for: Telemedicine interventions in six conflict-affected countries in the WHO Eastern Mediterranean region: a systematic review
Source: Confl Health. 2022 Dec 14;16:64. doi: 10.1186/s13031-022-00493-7 (PMC9748883; doi:10.1186/s13031-022-00493-7)
Supplement: Supplementary file 1 — Additional file 1. Academic database and grey literature search strategy. Full search strategy used for both academic database and grey literature search. [file 13031_2022_493_MOESM1_ESM.docx]

**Academic database and grey literature search strategy**

**Academic database search**

A search strategy was generated and duplicated for all academic databases and only modified where MESH terms differed. The following databases were searched with the OVID search engine: EMBASE, MEDLINE, MIDIRS, PsychInfo, Global Health, HMIC, CINAHL. In addition, the following databases were searched individually: Scopus, Web of Science, Cochrane Library. See table 1 for example of search strategy used for EMBASE database.

*Table 1: Example search strategy used to search EMBASE database*

| **Search strategy** | **EMBASE**  **Searched on 05/03/2020 -> 522 results** |
| --- | --- |
| 1 | (Iraq/ OR iraq* OR baghdad OR erbil OR basrah OR basra OR Syrian Arab Republic/ OR syria* OR aleppo OR damascus OR idlib OR Afghanistan/ OR afghan* OR kabul OR helmand OR Yemen/ OR yemen* OR sanaa OR sana’a OR Libyan Arab Jamahiriya/ OR libya* OR tripoli OR benghazi OR gaza* OR “gaza strip”) |
| 2 | (telemedicine/ OR exp telehealth/ OR tele* OR telenursing/ OR ehealth OR "e-health" OR "emental health" Or "e-mental health" OR "emedic*" OR "e-medic*" OR econsult* OR "e-consult*" OR ediagnos* OR "e-diagnos*" OR "video conferenc*" Or "mobile health" OR mhealth OR "m-health" OR videoconferencing/ OR videoconferenc* OR information technology/ OR information communication tech* OR information tech* OR (store adj2 forward) OR (Mobile adj2 tech*) OR Elearning OR "e-learning") |
| 3 | ((internet or digital* or "web-based" or provider or mobile or online or smartphone* or cell*phone* or telephone* or "mobile phone*" or phone* or "phone based" or "text-messag*") adj2 (treat* or intervention* or therap* or consult* or medic* or prescri* or diagnos* or care or manag*)) |
| 4 | ((mobile or distance) adj2 (educat* or training or learning)) |
| 5 | 2 OR 3 OR 4 |
| 6 | 1 AND 5 |
| 7 | limit to yr="2000-2020" |

**Grey literature search**

An exhaustive list of terms was used for grey literature search: telemedicine Syria, telemedicine Yemen, telemedicine Libya, telemedicine Iraq, telemedicine Afghanistan, telemedicine Gaza, telehealth Syria, telehealth Yemen, telehealth Libya, telehealth Iraq, telehealth Afghanistan, telehealth Gaza

Each phrase was searched individually in Google Scholar (scholar.google.com), World Health Organisation website (who.int) and Medecins Sans Frontiers website (msf.org.uk).
