# Supplementary material for: Telemedicine interventions in six conflict-affected countries in the WHO Eastern Mediterranean region: a systematic review
Source: Confl Health. 2022 Dec 14;16:64. doi: 10.1186/s13031-022-00493-7 (PMC9748883; doi:10.1186/s13031-022-00493-7)
Supplement: Supplementary file 2 — Additional file 2. Quality assessment of included studies. The method of quality appraisal of included studies is illustrated by the checklists used to assess quality and risk of bias, as well as data used to generate a quality score for each study. [file 13031_2022_493_MOESM2_ESM.docx]

**Quality assessment**

The Joanna Briggs Institute checklist for assessing risk of bias was used for descriptive reports [26]. The National Heart, Lung and Blood Institute’s quality assessment tools was used for observational and experimental studies [27]. Each domain for risk of bias was graded as ‘yes’ or ‘no’ or ‘other (cannot determine, not applicable, not reported)’. A score was calculated from the percentage of domains marked ‘yes’. Each study was categorised as low (≤49%), moderate (50–74%), or high (≥75%) quality.

Commentaries and case reports

*Table B.1: Domains in Joanna Briggs Institute checklist for quality assessment of commentaries and case reports*

| **Criteria** | **Yes** | **No** | **Other (CD, NA, NR)^a^** |
| --- | --- | --- | --- |
| - - - 1. Is the source of the opinion clearly identified? |  |  |  |
| - - - 1. Does source of opinion have standing in the field of expertise? |  |  |  |
| - - - 1. Are the interests of the relevant population the central focus of the opinion? |  |  |  |
| - - - 1. Is the stated position the result of an analytical process, and is there logic in the opinion expressed? |  |  |  |
| - - - 1. Is there reference to the extant literature? |  |  |  |
| - - - 1. Is any incongruence with the literature/sources logically defended? |  |  |  |
| **Total** |  |  |  |

*^a^CD – cannot determine, NA – not applicable, NR – not reported*

*Table B.2: Quality assessment scores of included articles that were commentaries or case reports*

| **Author and publication year** | **Domain** | | | | | | **Score (% domain filled)** |
| --- | --- | --- | --- | --- | --- | --- | --- |
|  | **1** | **2** | **3** | **4** | **5** | **6** |  |
| *Swinfen et al. 2005* | Y | Y | Y |  |  |  | Moderate (50) |
| *Olsen et al. 2010* | Y | Y | Y | Y |  |  | Moderate (66) |
| *Al-Makki et al. 2014* | Y |  | Y | Y | Y | Y | High (83) |
| *Sajwani et al. 2015* |  |  |  | Y | Y |  | Low (33) |
| *Jefee-Bahloul et al. 2016* | Y | Y | Y | Y | Y | Y | High (100) |
| *Moughrabieh et al. 2016* | Y |  | Y | Y | Y | Y | High (83) |
| *Al-Kamel et al. 2017* | Y | Y | Y | Y |  | Y | High (83) |
| *Alrifai et al. 2018* | Y |  | Y | Y | Y | Y | High (83) |

Case series

*Table B.3: Domains in National Heart, Lung and Blood Institute checklist for quality assessment of case series*

| **Criteria** | **Yes** | **No** | **Other (CD, NA, NR)^a^** |
| --- | --- | --- | --- |
| 1. Was the study question or objective clearly stated? |  |  |  |
| 2. Was the study population clearly and fully described, including a case definition? |  |  |  |
| 3. Were the cases consecutive? |  |  |  |
| 4. Were the subjects comparable? |  |  |  |
| 5. Was the intervention clearly described? |  |  |  |
| 6. Were the outcome measures clearly defined, valid, reliable, and implemented consistently across all study participants? |  |  |  |
| 7. Was the length of follow-up adequate? |  |  |  |
| 8. Were the statistical methods well-described? |  |  |  |
| 9. Were the results well-described? |  |  |  |
| **Total** |  |  |  |

*^a^CD – cannot determine, NA – not applicable, NR – not reported*

*Table B.4: Quality assessment scores of included articles that were case series*

| **Author and publication year** | **Domain** | | | | | | | | | **Score (% domain filled)** |
| --- | --- | --- | --- | --- | --- | --- | --- | --- | --- | --- |
|  | **1** | **2** | **3** | **4** | **5** | **6** | **7** | **8** | **9** |  |
| *Patterson et al. 2007* | Y | Y | Y | Y | Y |  | Y | Y | Y | High (89) |
| *Al-Hadad et al. 2011* | Y | Y | Y | Y |  |  |  |  |  | Low (44) |
| *Jefee-Bahloul et al. 2014* |  | Y | Y | Y | Y |  |  |  |  | Low (44) |
| *Khoja et al. 2017* |  |  | Y | Y |  |  |  |  |  | Low (22) |
| *Ghbeis et al. 2018* | Y |  | Y | Y | Y | Y | Y |  |  | Moderate (67) |
| *Ismail et al. 2018* | Y | Y | Y | Y | Y | Y | Y |  | Y | High (89) |
| *Masrani et al. 2018* | Y |  | Y | Y | Y |  |  |  | Y | Moderate (56) |
| *Belman et al. 2019* |  |  |  |  | Y | Y |  | Y |  | Low (33) |
| *Almoshmosh et al. 2020* | Y | Y | Y | Y | Y | Y | Y |  | Y | High (89) |
| *Rezaian et al. 2020* | Y | Y |  | Y | Y |  | Y |  |  | Moderate (56) |

*Y – fulfilled domain criteria*

Before-after interventions

*Table B.5: Domains in National Heart, Lung and Blood Institute checklist for quality assessment of before-after interventions*

| **Criteria** | **Yes** | **No** | **Other (CD, NA, NR)^a^** |
| --- | --- | --- | --- |
| 1. Was the study question or objective clearly stated? |  |  |  |
| 2. Were eligibility/selection criteria for the study population prespecified and clearly described? |  |  |  |
| 3. Were the participants in the study representative of those who would be eligible for the test/service/intervention in the general or clinical population of interest? |  |  |  |
| 4. Were all eligible participants that met the prespecified entry criteria enrolled? |  |  |  |
| 5. Was the sample size sufficiently large to provide confidence in the findings? |  |  |  |
| 6. Was the test/service/intervention clearly described and delivered consistently across the study population? |  |  |  |
| 7. Were the outcome measures prespecified, clearly defined, valid, reliable, and assessed consistently across all study participants? |  |  |  |
| 8. Were the people assessing the outcomes blinded to the participants' exposures/interventions? |  |  |  |
| 9. Was the loss to follow-up after baseline 20% or less? Were those lost to follow-up accounted for in the analysis? |  |  |  |
| 10. Did the statistical methods examine changes in outcome measures from before to after the intervention? Were statistical tests done that provided p values for the pre-to-post changes? |  |  |  |
| 11. Were outcome measures of interest taken multiple times before the intervention and multiple times after the intervention (i.e., did they use an interrupted time-series design)? |  |  |  |
| 12. If the intervention was conducted at a group level (e.g., a whole hospital, a community, etc.) did the statistical analysis take into account the use of individual-level data to determine effects at the group level? |  |  |  |
| **Total** |  |  |  |

*Table B.6: Quality assessment scores of included articles that were before-after interventions*

| **Author and publication year** | **Domain** | | | | | | | | | | | | **Score (% domain filled)** |
| --- | --- | --- | --- | --- | --- | --- | --- | --- | --- | --- | --- | --- | --- |
|  | **1** | **2** | **3** | **4** | **5** | **6** | **7** | **8** | **9** | **10** | **11** | **12** |  |
| *Ismail et al. 2018* | Y |  | Y |  |  | Y | Y |  |  |  |  |  | Low (33) |
| *Fritz et al. 2019* | Y | Y | Y |  |  | Y | Y |  |  | Y |  |  | Moderate (50) |

*Y – fulfilled domain criteria*

Cross-sectional studies

*Table B.7: Domains in National Heart, Lung and Blood Institute checklist for quality assessment of cross-sectional studies*

| **Criteria** | **Yes** | **No** | **Other (CD, NA, NR)^a^** |
| --- | --- | --- | --- |
| 1. Was the study question or objective clearly stated? |  |  |  |
| 2. Was the study population clearly specified and defined? |  |  |  |
| 3. Were all the subjects selected or recruited from the same or similar populations (including the same time period)? Were inclusion and exclusion criteria for being in the study prespecified and applied uniformly to all participants? |  |  |  |
| 4. Was the participation rate of eligible persons at least 50%? |  |  |  |
| 5. Was a sample size justification, power description, or variance and effect estimates provided? |  |  |  |
| 6. For the analyses in this paper, were the exposure(s) of interest measured prior to the outcome(s) being measured? |  |  |  |
| 7. Was the timeframe sufficient so that one could reasonably expect to see an association between exposure and outcome if it existed? |  |  |  |
| 8. For exposures that can vary in amount or level, did the study examine different levels of the exposure as related to the outcome (e.g., categories of exposure, or exposure measured as continuous variable)? |  |  |  |
| 9. Were the exposure measures (independent variables) clearly defined, valid, reliable, and implemented consistently across all study participants? |  |  |  |
| 10. Was the exposure(s) assessed more than once over time? |  |  |  |
| 11. Were the outcome measures (dependent variables) clearly defined, valid, reliable, and implemented consistently across all study participants? |  |  |  |
| 12. Were the outcome assessors blinded to the exposure status of participants? |  |  |  |
| 13. Was loss to follow-up after baseline 20% or less? |  |  |  |
| 14. Were key potential confounding variables measured and adjusted statistically for their impact on the relationship between exposure(s) and outcome(s)? |  |  |  |
| **Total** |  |  |  |

*Table B.8: Quality assessment scores of included articles that were cross-sectional studies*

| **Author and publication year** | **Domain** | | | | | | | | | | | | | | **Score (% domain filled)** |
| --- | --- | --- | --- | --- | --- | --- | --- | --- | --- | --- | --- | --- | --- | --- | --- |
|  | **1** | **2** | **3** | **4** | **5** | **6** | **7** | **8** | **9** | **10** | **11** | **12** | **13** | **14** |  |
| *Abd Ghani et al. 2015* | Y |  |  |  | Y |  | Y |  |  |  | Y |  |  |  | Low (28) |
| *Sayani et al. 2019* | Y | Y |  | y |  | Y | Y | Y | Y |  | Y |  |  | Y | Moderate (64) |

*Y – fulfilled domain criteria*

Randomised control trials

*Table B.9: Domains in National Heart, Lung and Blood Institute checklist for quality assessment of randomised control trials*

| **Criteria** | **Yes** | **No** | **Other (CD, NA, NR)^a^** |
| --- | --- | --- | --- |
| 1. Was the study described as randomized, a randomized trial, a randomized clinical trial, or an RCT? |  |  |  |
| 2. Was the method of randomization adequate (i.e., use of randomly generated assignment)? |  |  |  |
| 3. Was the treatment allocation concealed (so that assignments could not be predicted)? |  |  |  |
| 4. Were study participants and providers blinded to treatment group assignment? |  |  |  |
| 5. Were the people assessing the outcomes blinded to the participants' group assignments? |  |  |  |
| 6. Were the groups similar at baseline on important characteristics that could affect outcomes (e.g., demographics, risk factors, co-morbid conditions)? |  |  |  |
| 7. Was the overall drop-out rate from the study at endpoint 20% or lower of the number allocated to treatment? |  |  |  |
| 8. Was the differential drop-out rate (between treatment groups) at endpoint 15 percentage points or lower? |  |  |  |
| 9. Was there high adherence to the intervention protocols for each treatment group? |  |  |  |
| 10. Were other interventions avoided or similar in the groups (e.g., similar background treatments)? |  |  |  |
| 11. Were outcomes assessed using valid and reliable measures, implemented consistently across all study participants? |  |  |  |
| 12. Did the authors report that the sample size was sufficiently large to be able to detect a difference in the main outcome between groups with at least 80% power? |  |  |  |
| 13. Were outcomes reported or subgroups analyzed prespecified (i.e., identified before analyses were conducted)? |  |  |  |
| 14. Were all randomized participants analyzed in the group to which they were originally assigned, i.e., did they use an intention-to-treat analysis? |  |  |  |
| **Total** |  |  |  |

*Table B.10: Quality assessment scores of included articles that were randomised control trials*

| **Author and publication year** | **Domain** | | | | | | | | | | | | | | **Score (% domain filled)** |
| --- | --- | --- | --- | --- | --- | --- | --- | --- | --- | --- | --- | --- | --- | --- | --- |
|  | **1** | **2** | **3** | **4** | **5** | **6** | **7** | **8** | **9** | **10** | **11** | **12** | **13** | **14** |  |
| *Wagner et al. 2012* | Y |  |  |  |  | Y |  |  |  |  | Y |  | Y |  | Low (29) |
| *Knaevelsrud et al. 2015* | Y | Y |  |  |  | Y |  |  |  |  | Y |  | Y |  | Low (36) |

*Y – fulfilled domain criteria*
